# Nutrition Programs and Policies for Maternal and Child Health

**DOI:** 10.3390/nu17152397

**Published:** 2025-07-22

**Authors:** Blessing Akombi-Inyang

**Affiliations:** School of Population Health, Faculty of Medicine and Health, University of New South Wales, Sydney, NSW 2052, Australia; b.akombi@unsw.edu.au; Tel.: +61(2)-9385-8687

The Special Issue *Nutrition Programs and Policies for Maternal and Child Health* brings together a compelling body of work addressing some of the most pressing nutritional challenges facing mothers and children in low- and middle-income countries. This collection of five studies spans diverse geographies—from South Asia and Northern Africa to Southeast Asia and West Africa—and provides nuanced insights into both the biological and socio-political dimensions of maternal and child nutrition. Together, these papers contribute valuable evidence for the formulation and implementation of equitable, context-sensitive nutrition policies and interventions.

Agho et al. [1] examined the intersection of child undernutrition and iron deficiency in Nepal, using nationally representative data from 1702 children aged 6–59 months. The study found a high prevalence of stunting (35.6%), underweight (29.0%), and wasting (11.7%), with significant associations between stunting and anemia, as well as iron deficiency. Children aged 6–23 months were at especially high risk. The findings underscore the urgent need for integrated interventions targeting undernutrition to mitigate the widespread burden of iron deficiency and its developmental consequences.

Nguyen et al. [2] investigated the sharp decline in early initiation of breastfeeding (EIBF) practices in Vietnam, reporting a prevalence of just 25.5% among mothers of children under 24 months. Cesarean delivery was found to be a strong negative predictor of EIBF, reducing the odds by 75%, alongside younger maternal age and lack of immediate skin-to-skin contact. This study calls for renewed efforts to reinforce breastfeeding promotion, especially among young mothers and those delivering via cesarean section and highlights the importance of supportive clinical protocols during childbirth.

Elmighrabi, Fleming, and Agho [3] presented an extensive analysis of wasting and underweight among 37,816 children across Algeria, Egypt, Sudan, and Tunisia. The study revealed regional disparities, with Sudan bearing the greatest burden. Socioeconomic disadvantages, limited maternal education, and inadequate dietary diversity were among the significant predictors. These findings point to the critical need for region-specific strategies that combine poverty alleviation, maternal education, and improved access to diverse foods to address persistent undernutrition in Northern Africa.

A randomized controlled pilot trial by Jannat et al. [4] explored the potential benefits of combining yogurt supplementation with nutritional education in Dhaka’s urban slums. While the intervention did not result in statistically significant improvements in linear growth over three months, the yogurt group showed a positive trend in growth and dietary diversity. These preliminary results suggest potential for further research into affordable, culturally acceptable food-based interventions to support child nutrition in poor urban areas.

Finally, Bastos-Moreira et al. [5] outlined a multi-omics protocol nested within a trial in Burkina Faso, assessing the impact of balanced energy–protein (BEP) supplementation on maternal and newborn health. While the paper is a study protocol, its innovative approach—integrating human biomonitoring and multi-omics—promises to deliver granular insights into the biological pathways influenced by nutritional interventions during pregnancy and lactation. This systems-level perspective is crucial for optimizing future maternal and child nutrition programming.

In closing, this Special Issue highlights the multifaceted nature of nutritional challenges affecting maternal and child health. Each contribution not only advances the evidence base but also reinforces the importance of context-specific, equitable nutrition strategies. We extend our appreciation to the contributing authors and reviewers whose diligent efforts have shaped this important collection. It is our hope that the insights shared here will inform future research, policy development, and implementation that better support the health and well-being of women and children globally.
